# Recovery of complete left bundle branch block in a dilated cardiomyopathy patient after treatment with sacubitril/valsartan: A case report

**DOI:** 10.1097/MD.0000000000029330

**Published:** 2022-07-08

**Authors:** Meng-Ling Peng, Yu Fu, Ying Zhang, Chu-Wen Wu, Hang Ren, Shan-Shan Zhou

**Affiliations:** a Department of Cardiology, The First Hospital of Jilin University, Chaoyang District, Changchun, People’s Republic of China; b Department of Cardiology, The Second Hospital of Jilin University, Changchun, People’s Republic of China.

**Keywords:** complete left bundle branch block, dilated cardiomyopathy, left ventricular reverse remodeling, normal electrocardiogram

## Abstract

**Rationale:**

The treatment of dilated cardiomyopathy (DCM) has recently been greatly improved, especially with the widespread use of sacubitril/valsartan (ARNI) combination therapy. We know that ARNI-like drugs can significantly improve the symptoms of heart failure with reducing ejection fraction. However, clinical studies evaluating the safety and efficacy of ARNI in DCM-associated arrhythmia are limited, and whether individuals with arrhythmia would benefit from ARNI remains controversial. In this case, we report a patient with complete left bundle branch block (CLBBB) associated with DCM whose CLBBB returned to normal after treatment with ARNI.

**Patient concerns:**

A 38-year-old man was admitted to the hospital for 20 days for idiopathic paroxysmal dyspnea. He presented with exacerbated dyspnea symptoms at night, accompanied by cough and sputum.

**Diagnosis:**

Physical examination revealed a grade 4/6 systolic murmur could be heard in the apical area of the heart and mild edema was present in both lower limbs. Laboratory examination found that the B-type natriuretic peptide was significantly increased. Echocardiography indicated left atrial internal diameter, right ventricular internal diameter, and left ventricular diastolic diameter were enlarged and ejection fraction was significantly decreased. Besides, the pulsation of the wall was diffusely attenuated. Electrocardiogram was suggestive of tachycardia and CLBBB. A diagnosis of DCM with CLBBB was considered based on a comprehensive evaluation of the physical examination, laboratory examination, echocardiography and electrocardiogram.

**Interventions:**

The patient was treated with ARNI at a dose of 50 mg (twice a day) at first, gradually increasing to the target dose (200 mg, twice a day) in the following 9 months as shown in Table 1, along with metoprolol 25 mg (once a day [qd]), diuretics 20 mg (qd), and aldosterone 20 mg (qd).

**Outcomes:**

After treatment with ARNI during the 9-month follow-up, the patient’s symptoms improved, and CLBBB returned to normal.

**Lessons:**

Clinical studies evaluating the safety and efficacy of ARNI in DCM-associated arrhythmia are limited, and whether individuals with arrhythmia would benefit from ARNI remains controversial. This report will help to instruct the clinical treatment of DCM patients with CLBBB and the potential application of ARNI.

## 1. Introduction

Dilated cardiomyopathy (DCM) is a heterogeneous form of cardiomyopathy characterized by left ventricular or biventricular enlargement with systolic dysfunction.^[1]^ DCM is a common cause of heart failure (HF), arrhythmia, and sudden death. Inhibiting activation of the sympathetic nervous system, suppressing the renin–angiotensin–aldosterone system (RAAS), and improving imbalances of the natriuretic peptide system are important strategies for delaying the progression of DCM.^[2]^ Based on this mechanism, sacubitril/valsartan (ARNI) improves HF by reducing ejection fraction (EF). Drugs such as angiotensin receptor-1 enkephalin inhibitors can delay and reverse ventricular remodeling, and furthermore, it can reduce hospitalization and mortality of HF.^[3]^ However, clinical studies evaluating the safety and efficacy of ARNI in DCM-associated arrhythmia are limited, and whether individuals with arrhythmia would benefit from ARNI remains controversial. Due to DCM-associated degeneration and fibrosis of the conduction system, poor ventricular remodeling, and ischemia, complete left bundle branch block (CLBBB) can occur in DCM patients. This article reports a case of DCM with severe HF and CLBBB. After treatment with ARNI during the 9-month follow-up, the patient’s symptoms improved, and CLBBB returned to normal. This report will help to instruct the clinical treatment of DCM patients with CLBBB and the potential application of ARNI.

## 2. Case report

The patient, a 38-year-old man, was admitted to the hospital for 20 days for idiopathic paroxysmal dyspnea. He presented with exacerbated dyspnea symptoms at night, accompanied by cough and sputum, with no accompanying abdominal pain. The patient did not receive any treatment. The patient was previously physically fit.

Clinical signs on admission: temperature 36.5°C, pulse 78 beats/min, respiration 16 breaths/min, blood pressure 111/63 mm Hg, clear consciousness, and cooperative examination. The patient’s skin and sclera were not jaundiced, and he had a regular heart rhythm, but a grade 4/6 systolic murmur could be heard in the apical area of the heart and mild edema was present in both lower limbs.

**Figure 1.**
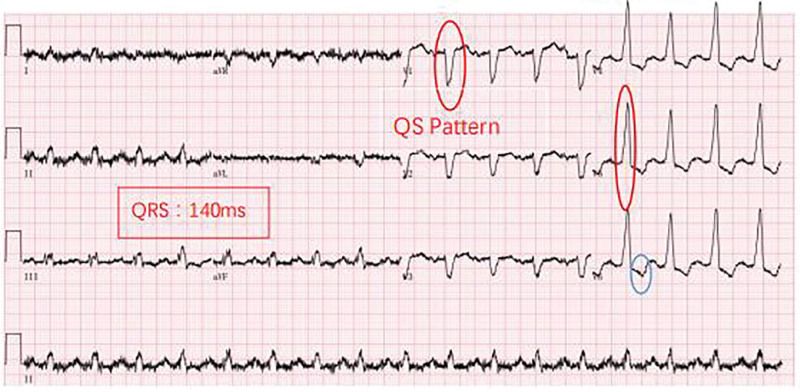
Pretreatment ECG demonstrating sinus tachycardia/CLBBB/ST-T changes/QRS: 140 ms/PR: 176 ms/HR: 103 bpm. bpm = breath per minute, CLBBB = complete left bundle branch block, ECG = electrocardiogram, HR = heart rate.

The patient’s electrocardiogram (ECG; Fig. 1) was suggestive of tachycardia and CLBBB. Echocardiography revealed left atrial diameter (LAD 54 mm, right ventricular internal diameter (RVD) 34 mm, septal thickness 9 mm, left ventricular diastolic diameter (LVDD) 71 mm, left ventricular posterior wall thickness 8 mm, right atrial internal diameter 50 × 61 mm, EF 23%, and diffusely attenuated pulsation of the wall. The patient did not undergo magnetic resonance imaging due to tachycardia. Laboratory examination revealed B-type natriuretic peptide 998.0 pg/mL (0–100 pg/mL), troponin 0.10 ng/mL (0–0.05 ng/mL), D-dimer 251 ng/mL (100–600 ng/mL), hemoglobin 179 g/L (130–175 g/L), leukocytes 10.18 × 10^9^/L (3.50–9.50 × 10^9^/L), platelets 355 × 10^9^ L (125–350 × 10^9^/L), and creatinine 98.9 µm (57–97 µm). Coronary angiography did not indicate coronary stenosis or collateral circulation.

The patient was diagnosed as having DCM with CLBBB. His condition was categorized as New York Heart Association functional class III. ARNI therapy was initiated following diagnosis. Drugs such as antiventricular remodeling inhibitors and diuretics were administered. The patient was discharged from the hospital with improved symptoms after 7-day treatment.

**Figure 2.**
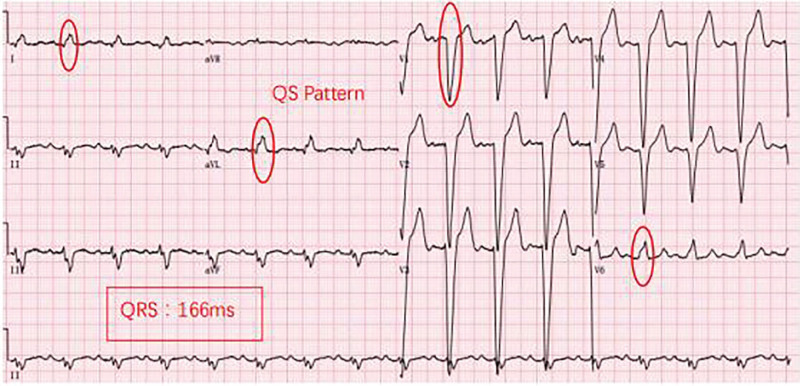
Month 1 ECG revealing left ventricular hypertrophy and ST-T changes/HR: 97 bpm/PR: 180 ms/QRS: 166 ms. bpm = breath per minute, ECG = electrocardiogram, HR = heart rate.

**Figure 3.**
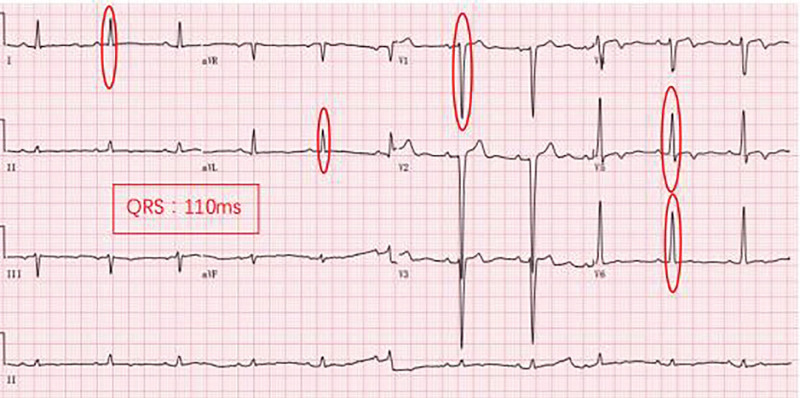
Month 2 ECG revealing sinus/left ventricular hypertrophy and ST-T changes/HR: 67 bpm/QRS: 110 ms. bpm = breath per minute, ECG = electrocardiogram, HR = heart rate.

**Figure 4.**
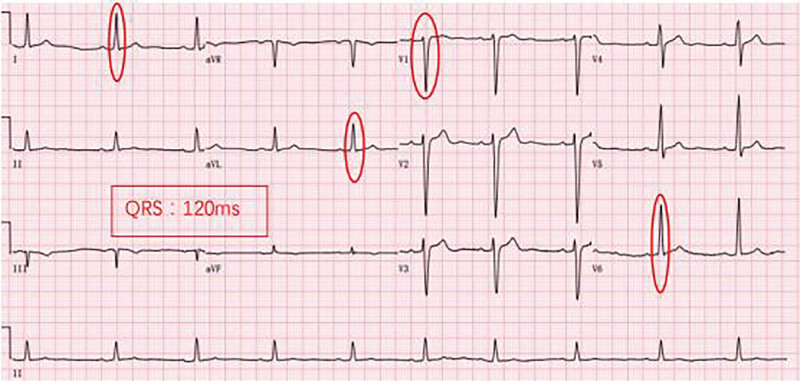
Month 4 ECG revealing sinus bradycardia and left ventricular hypertrophy/HR: 59 bpm/PR: 182 ms/QRS: 120 ms. bpm = breath per minute, ECG = electrocardiogram, HR = heart rate.

**Figure 5.**
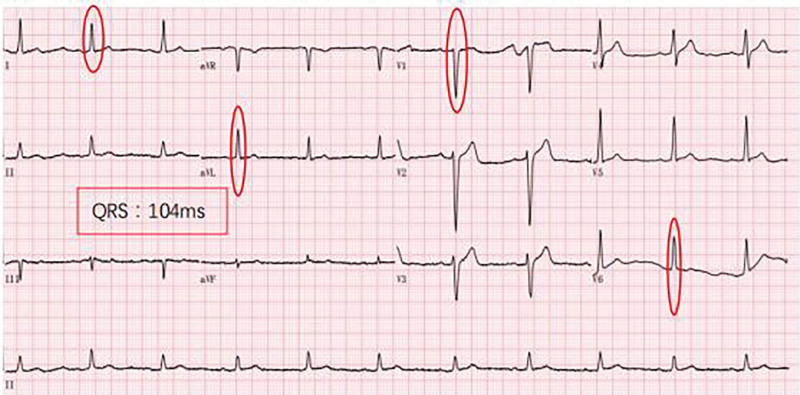
Month 8 ECG revealing sinus rhythm/HR: 65 bpm/PR: 170 ms/QRS: 104 ms. bpm = breath per minute, ECG = electrocardiogram, HR = heart rate.

The patient was treated with ARNI at a dose of 50 mg (twice a day) at first, gradually increasing to the target dose (200 mg, twice a day) in the following 9 months, along with metoprolol 25 mg (once a day [qd]), diuretics 20 mg (qd), aldosterone 20 mg (qd). The patient’s cardiac ultrasound ECG was rechecked 1, 2, 4, and 8 months after initiating treatment. ECG was still CLBBB at month 1 postdischarge (Fig. 2) while CLBBB was no longer evident at month 2 postdischarge (Fig. 3). Further, ECGs revealed no trace of CLBBB on months 4 and 8 postdischarge (Figs. 4 and 5). With no significant change in PR interval and a significantly shorter QRS time, the LVDD gradually decreased from 71 to 59 mm (Table 2), and the EF increased from 23% to 47%, the LAD decreased from 54 to 37 mm, the RVD decreased from 34 to 24 mm, and his condition was classified as New York Heart Association class I after 9 months. The follow-up lasted for 9 months, and the patient is still undergoing intermittent reexamination and continuous medication.

**Table 1:** Specific medications used in treatment.

Month(s) and dates after discharge	Morning (ARNI)	Night (ARNI)	Metoprolol 23.75 mg QD; diuretics (furosemide) 20 mg QD; aldosterone 20 mg QD
Month 1 (28/02/20–27/03/20)	50 mg	50 mg	
Month 2 (27/03/20–26/04/20)	100 mg	50 mg	
Month 3–4 (26/04/20–28/06/20)	100 mg	100 mg	
Months 5–7 (28/05/20–29/08/20)	150 mg	100 mg	
Months 8–11 (29/08/20–13/11/20)	150 mg	150 mg	
Month 11–present (13/11/20–)	200 mg	200 mg	

ARNI = sacubitril/valsartan, QD = once a day.

**Table 2:** Echocardiography changes by date.

Parameter	28/2/20	27/3/20	26/4/20	28/6/20	13/11/20
EF (%)	23	23	33	44	47
FS (%)	11	11	16	22	27
LVDD (mm)	71	70	71	57	59
LAD (mm)	54	50	41	39	37
RVD (mm)	34	25	20	25	24

EF = ejection fraction, FS = left ventricular fractional shortening rate, LAD = left atrial diameter, LVDD = left ventricular diastolic diameter, RVD = right ventricular diameter.

## 3. Discussion

DCM is characterized by LV dilatation and contractile dysfunction in the absence of abnormal loading conditions or severe coronary artery disease. The most common presenting symptoms are related to congestive HF, but can also include circulatory collapse, arrhythmias, and thromboembolic events. The progression and prognosis of DCM are primarily influenced by the degree of LV dilatation.^[1]^

LBBB is an independent predictor of adverse events and outcomes in DCM, which include sudden cardiac death, mortality due to HF, and myocardial infarction.^[4,5]^ We found that LBBB induction caused decreased left ventricular EF (LVEF), increased LVDD and wall mass, and decreased reduced septal perfusion.^[6]^ These findings suggested that LBB-related electromechanical dyssynchrony could play an important role in progression of DCM.

The cellular pathology DCM is characterized by cardiomyocyte hypertrophy and degeneration, myocardial interstitial fibrosis, ventricular cavity dilation, and thinning of the ventricular wall with fibrous scar formation.^[1]^ There is a large potential electrical gradient between the fibrotic and normal myocardium, resulting in significant heterogeneity in excitability and conduction in different parts of the myocardium. This heterogeneity in conductivity causes uncoordinated cardiac contraction and arrhythmias. In addition, enlargement of the heart causes excessive elongation and potential rupture of the conduction system, resulting in delayed and blocked conductivity.

Therefore, the conventional treatment for DCM patients with CLBBB is cardiac resynchronization therapy (CRT), also known as biventricular pacing.^[7,8]^ CRT can reverse left ventricular dyssynchrony in a process referred to as left ventricular reverse remodeling (LVRR).^[9,10]^ In combination with appropriate pharmacologic interventions, CRT improves survival, reduces hospital admissions, and induces reverse left ventricular remodeling in appropriately selected patients. However, the use of CRT is limited due to its high cost and relatively high failure rate of 30%.^[11]^ Therefore, optimized treatment plans such as ARNI are of great significance in patients with DCM and CLBBB.

ARNI is an angiotensin receptor-enkephalinase inhibitor and is used in HF patients with reduced EF based on the results of the prospective comparison of ARNI with ACEI to determine impact on global mortality and morbidity in heart failure trial trial.^[3]^ In addition to modulating the RAASes sympathetic tone through its diuretic effects.^[2,12]^ However, the potential antiarrhythmic effects of ARNI have not been elucidated.

We postulated that the ability of ARNI to reverse pathological myocardial remodeling could indirectly decrease ventricular arrhythmias by improving uneven and slow conduction associated with remodeling.^[2]^ Currently available experimental studies do not support a direct antiarrhythmic effect. A large-scale real-world clinical trial demonstrated that ARNI attenuated electrical and structural remodeling in the context of atrial fibrillation (AF), possibly by inhibiting T-cell activation and calcium-regulated phosphatase, subsequently attenuating AF.^[13]^ Therefore, for HF patients with AF, ARNI may reduce the occurrence of AF by reversing electrical and structural remodeling of the heart. ARNI likely improves HF symptoms by simultaneously modulating the RAAS and sympathetic nervous system. HF patients with AF may benefit more from simultaneous treatment with AF therapies and ARNI.

In the present case study, the patient’s CLBBB disappeared, which is extremely rare. We suggest that this could be due to 2 possible mechanisms. First, as lesions develop, the right bundle branch could develop the same degree of conduction block or delay. If this is the case, the patient will evolve from CLBBB to atrioventricular block type I. Second, DCM-triggering factors could be improved by ARNI. For example, if myocardial ischemia is improved, ventricular remodeling would be controlled, and the ventricle would decrease in size. These events, or a combination thereof, could cause CLBBB to intermittently return to normal. In this case, CLBBB resolution was concomitant with improved symptoms, decreased LVDD, and improved LVEF. The patient did not present with prolonged PR interval. Given these findings, we suggest that the second possibility, wherein ARNI alleviates DCM triggers, is more likely. Therefore, we hypothesize that ARNI could suppress pathological ventricular remodeling or even LVRR in DCM. LVRR is defined as reduced LVDD and improved LVEF.^[14]^ LVRR is a complex process involving remodeling of the LV and many other cardiac structures, as observed in our follow-up. LVRR is considered to be an important prognostic tool in the treatment of DCM.^[13–15]^ However, many studies of LVRR are ongoing, including the optimal timing of LVRR quantification and the background of individual genetics.^[13,14]^ LVRR is estimated to occur in up to 40% of patients with DCM, suggesting that DCM is not necessarily an irreversible progressive myocardial disease. This implies that LVRR is a significant therapeutic target for DCM.

Despite novelty and potential clinical relevance of this study, its limitations should be acknowledged to avoid its overinterpretation. During treatment of the patient, cardiac magnetic resonance imaging was performed too late to develop a strong baseline. The patient was also not advised to undergo cardiac magnetic resonance imaging during subsequent follow-up, so we were unable to obtain more intuitive imaging data to evaluate the improvement of cardiac fibrosis. This deficiency will be addressed in future follow-up studies.

In summary, the present case demonstrated that ARNI could improve DCM prognosis, reverse ventricular remodeling, improve uneven electrical conduction, and improve CLBBB. Future clinical studies will evaluate the further application of ARNI in cardiac arrhythmias.

## Acknowledgments

The authors would like to thank the patient and her family for granting us their permission to publish this case report.

## Author contributions

Conceptualization: Meng-Ling Peng.

Data collection and curation: Meng-Ling Peng, Yu Fu, Ying Zhang.

Methodology: Yu Fu, Ying Zhang, Chu-Wen Wu.

Writing – original draft: Meng-Ling Peng, Yu Fu, Chu-Wen Wu.

Writing – review & editing: Meng-Ling Peng, Hang Ren, Shan-Shan Zhou.
